# Evolution of the *DAZ *gene and the *AZFc *region on primate Y chromosomes

**DOI:** 10.1186/1471-2148-8-96

**Published:** 2008-03-26

**Authors:** Yueh-Hsiang Yu, Yi-Wen Lin, Jane-Fang Yu, Werner Schempp, Pauline H Yen

**Affiliations:** 1 Graduate Institute of Life Sciences, National Defense Medical Center, Taipei, Taiwan; 2 Institute of Biomedical Sciences, Academia Sinica, Taiwan; 3 Taipei Zoo, Taipei, Taiwan; 4 Institute of Human Genetics and Anthropology, University of Freiburg, Germany

## Abstract

**Background:**

The *Azoospermia Factor c *(*AZFc*) region of the human Y chromosome is a unique product of segmental duplication. It consists almost entirely of very long amplicons, represented by different colors, and is frequently deleted in subfertile men. Most of the *AZFc *amplicons have high sequence similarity with autosomal segments, indicating recent duplication and transposition to the Y chromosome. The *Deleted in Azoospermia *(*DAZ*) gene within the red-amplicon arose from an ancestral autosomal *DAZ-like *(*DAZL*) gene. It varies significantly between different men regarding to its copy number and the numbers of RNA recognition motif and DAZ repeat it encodes. We used Southern analyses to study the evolution of *DAZ *and *AZFc *amplicons on the Y chromosomes of primates.

**Results:**

The Old World monkey rhesus macaque has only one *DAZ *gene. In contrast, the great apes have multiple copies of *DAZ*, ranging from 2 copies in bonobos and gorillas to at least 6 copies in orangutans, and these *DAZ *genes have polymorphic structures similar to those of their human counterparts. Sequences homologous to the various *AZFc *amplicons are present on the Y chromosomes of some but not all primates, indicating that they arrived on the Y chromosome at different times during primate evolution.

**Conclusion:**

The duplication and transposition of *AZFc *amplicons to the human Y chromosome occurred in three waves, i.e., after the branching of the New World monkey, the gorilla, and the chimpanzee/bonobo lineages, respectively. The red-amplicon, one of the first to arrive on the Y chromosome, amplified by inverted duplication followed by direct duplication after the separation of the Old World monkey and the great ape lineages. Subsequent duplication/deletion in the various lineages gave rise to a spectrum of *DAZ *gene structure and copy number found in today's great apes.

## Background

**Figure 1:**
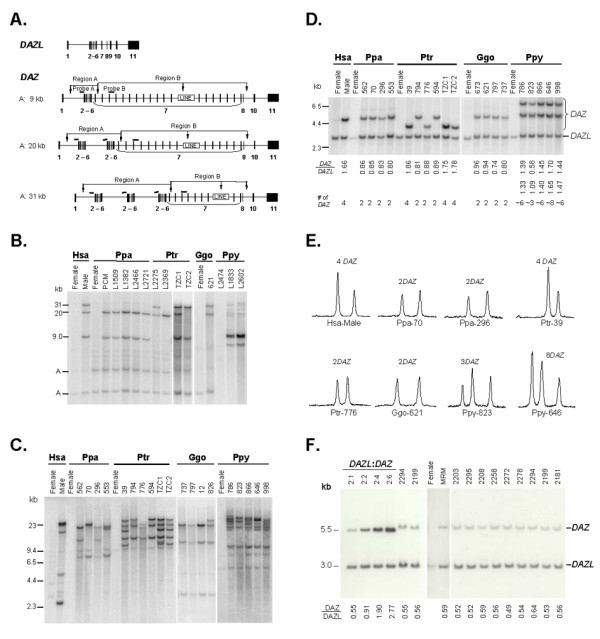
**Southern analysis of the *DAZ *genes on the Y chromosomes of great apes**. (A) Genomic structures of the human *DAZL *and *DAZ *genes. Exons 2–6 encode the RNA recognition motif and exon 7 encodes the DAZ repeat. Three *DAZ *genes encoding one, two, and three RRMs, respectively, are shown. NsiI sites are indicated with vertical arrows, and the sizes of NsiI fragments detected by probe A are given at left. (B) Southern analysis of Region A – the RRM blot. Genomic DNA samples from humans (Hsa), bonobos (Ppa), chimpanzees (Ptr), gorillas (Ggo), and orangutans (Ppy) were digested with NsiI and blotted with probe A. The autosomal *DAZL *fragments are indicated with A's at left. (C) Southern analysis of Region B – the DAZ repeat blot. The blots were hybridized with probe B that detects all exon 7 repeating units. (D) Determination of the *DAZ *gene copy number – the DAZ dosage blot. The probe contained a mixture of the human *DAZ *and *DAZL *3'UTRs. The human male sample contains 4 *DAZ *genes. The relative intensities of the *DAZ *and *DAZL *signals and the derived *DAZ *gene copy numbers are listed underneath the blots. Due to reduced similarity with the human probe and the high copy number, the copy numbers of orangutan *DAZ *gene could not be determined accurately. (E) Quantification of the hybridization signals of the DAZ dosage blots in (D). The *DAZ *peaks are at the left. (F) Determination of the *DAZ *gene copy number in rhesus macaque. The left four lanes contain *E. coli *DNA samples spiked with known amounts of rhesus macaque *DAZ *and *DAZL *3'UTRs. (See Methods for details.) The probe contains a mixture of rhesus macaque *DAZ *and *DAZL *3'UTRs. This figure only shows representative samples with different hybridization patterns. The results of all samples are described in Supplementary Table S1 (see Additional file 1).

Segmental duplications, also known as low copy repeats, constitute about 5% of the human genome (reviewed in [1]). They are thought to have arisen through distinct waves of duplication during evolution, and play important roles in creating new genes and shaping the genome. In addition, they contribute significantly to the instability of the genome and genetic variation in the human populations. A good example of segmental duplication is the *Deleted in Azoospermia (DAZ) *gene family that encodes germ-cell specific RNA-binding proteins implicated in the regulation of protein synthesis (reviewed in [2,3]). The family consists of three members, *BOULE*, *DAZL*, and *DAZ. BOULE *is found widely in the animal kingdom from worms to humans, *DAZL *is only present in vertebrates, and *DAZ *is only present in Old World monkeys, great apes and humans [4-8]. In humans, both *BOULE *and *DAZL *are located on autosomes as single-copy genes, whereas *DAZ *is present in multiple copies on the Y chromosome. Structurally, *BOULE *and *DAZL *encode a single RNA recognition motif (RRM) and one copy of a 24 amino acid repeat unit, dubbed the DAZ repeat, whereas the *DAZ *genes encode proteins with one, two, or three copies of RRM and a highly polymorphic DAZ repeat region that contains from 8 to 24 copies of the DAZ repeat (Figure 1A). It was suggested that *BOULE*, the ancestral copy of the gene family, duplicated and transposed to another autosome in an ancestor of the vertebrates to generate the *DAZL *gene, which again duplicated and transposed to the Y chromosome before the branching of the Old World monkey lineage to give rise to the *DAZ *genes [5,8,9].

**Figure 2:**
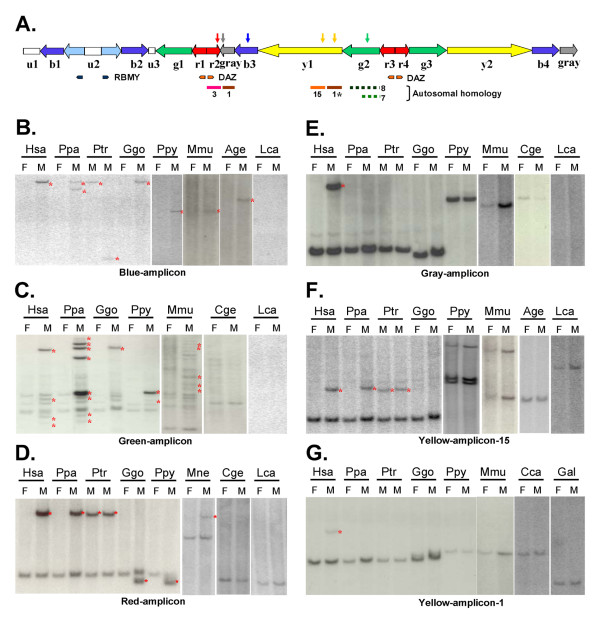
**Evolution of the *AZFc *amplicons in the primate lineage**. (A) The prototype architecture of the *AZFc *region on the human Y chromosome. The amplicons are color-coded according to [10]. The locations of the hybridization probes within the various amplicons are indicated above with vertical arrows, and the *DAZ *and the *RBMY *genes are indicated underneath. Autosomal homologous regions are shown at the bottom as colored horizontal lines with the chromosome numbers indicated underneath. Broken lines represent discontinuous homology. 1* indicates that in addition to chromosome 1, several other autosomes also share homology with the region. (B) – (G) Southern analyses of the presence of sequences homologous to the various amplicons on primate Y chromosomes. Human (Hsa), bonobo (Ppa), chimpanzee (Ptr), gorilla (Ggo), orangutan (Ppy), rhesus monkey (Mmu), pig-tail macaque (Mne), spider monkey (Age), marmoset (Cge), capuchin (Cca), galago (Gal) and lemur (Lca) genomic DNAs were digested with either HindIII or NsiI and hybridized with probes from the various amplicons as indicated. Yellow-amplicon-15 and yellow-amplicon-1 contain sequences homologous to chromosome 15 and chromosome 1, respectively. The Y chromosome fragments are marked with red asterisks. F: female, M: male.

The human *DAZ *genes are located on the Y chromosome long arm, within the red amplicons in the *Azoospermia Factor c *(*AZFc*) region which is frequently deleted in azoospermic men [10]. The *AZFc *region consists almost entirely of very long amplicons (Figure 2A) that are thought to have been acquired through three molecular evolutionary processes: persistence of sequences previously shared with the X chromosome, duplication and transposition of autosomal sequences, and retroposition of autosomal encoded mRNAs [10]. *AZFc *is one of the most polymorphic regions in the human genome, with many *AZFc *architectures and *DAZ *gene structures being reported [11-13]. Most men have four *DAZ *genes, with two encoding one RRM and one each encoding two and three RRMs (Figure 1A). However, a significant fraction of men in various populations have 2 or 6 *DAZ *genes, resulting from deletion/duplication through homologous recombination between the various amplicons (reviewed in [14]). It is not known whether such polymorphisms are also present in other primates. We used genomic Southern analyses to study the structures and the copy numbers of the *DAZ *genes in great apes and Old World monkeys. In addition, we also searched for *AZFc *amplicon homologous sequences on the Y chromosomes of primates. Our results allowed us to define the times when the various amplicons arrived on the Y chromosome and provide insights into the evolutionary history of the *AZFc *region.

## Results

### Southern analyses of the DAZ genes

The human *DAZ *genes encode different copies of RRM (encoded by exons 2–6) and DAZ repeat (encoded by exon 7) (Figure 1A). We previously designed two Southern hybridization blots, taking advantage of several nicely located NsiI restriction sites within the genes, to study these variable regions in a genomic DNA sample [16]. In the RRM blot, probe A from *DAZ *intron 1 detects a 9.0 kb, a 20 kb, or a 31 kb fragment when the *DAZ *gene contains one, two, or three copies of the RRM coding region, respectively. In the DAZ-repeat blot, probe B from intron 7 detects fragments of different sizes when the genes encode different copies of the DAZ repeats. The locations of the NsiI sites flanking the RRM region and the DAZ repeat region are conserved in the available chimpanzee sequence, and appears to be so in other great apes as well. These two blots allowed us to study the structure of the *DAZ *genes. We also developed a DAZ dosage blot, using the autosomal *DAZL *gene as an internal dosage standard, to determine the *DAZ *gene copy number [17]. Comparing the ratio of the hybridisation signals of the *DAZ *and *DAZL *fragments of a test sample to those of samples with known *DAZ *copy numbers provides the *DAZ *copy number of the test sample. We used these three Southern blots to analyse DNA samples from a collection of male great apes, including 10 bonobos (*Pan paniscus*, Ppa), 8 chimpanzees (*Pan troglodytes*, Ptr), 5 gorillas (*Gorilla gorilla*, Ggo), and 16 orangutans (*Pongo pygmaeus*, Ppy) (see Additional file 1). The animals were housed in different zoos in three continents and several of them were born in the wild. When the family trees were available, we selected only one male from each family to increase the chance that our animals of the same species carried different Y chromosomes.

We characterized 10 bonobo samples and all of them gave the same 20 kb and 9 kb Y-linked fragments on the RRM blots (Figure 1B; Additional file 1), and showed fragments of various sizes on the DAZ-repeat blots (Figure 1C). On the DAZ dosage blots they all gave a 4.7 kb *DAZ *fragment and a 3.0 kb *DAZL *fragment, with a relative intensity consistent with 2 *DAZ *genes (Figure 1D, 1E). Thus all the bonobos had one *DAZ *gene encoding one RRM and another one encoding two RRMs, and their DAZ repeat regions were polymorphic, similar to those observed in the human *DAZ *genes. The situation in chimpanzees was quite different. Of the 8 chimpanzees studied, five had two *DAZ *genes and three had 4 *DAZ *genes (Figure 1D, 1E; Additional file 1), and they gave different patterns on the RRM blots (Figure 1B; Additional file 1). Those with two *DAZ *genes (L2275, L2369, 594, 776 and 794) had either one or both of the two larger fragments at 31 and 20 kb, whereas those with four *DAZ *genes had all three fragments at 31, 20, and 9 kb (39 and TZC1), or only two fragments at 31 and 9 kb (TZC2). The DAZ-repeat blots showed again the polymorphic nature of the DAZ-repeat regions (Figure 1C). Thus the chimpanzees have two or four *DAZ *genes that encode various numbers of RRMs and DAZ repeats. The five gorilla studied all have 2 *DAZ *genes (Figure 1D, 1E) that gave the same 31 kb and 20 kb fragments on the RRM blots (Figure 1B) and various size fragments on the DAZ-repeat blots (Figure 1C). The 16 orangutans characterized included both Bornean orangutan (*Pongo pygmaeus pygmaeus*) and Sumatran orangutan (*Pongo pygmaeus abelii*) (Additional file 1). They gave a major 9 kb fragment and several minor fragments on the RRM blots (Figure 1B), and numerous large fragments of various lengths and intensities on the DAZ-repeat blots (Figure 1C). On the DAZ dosage blots, they gave two *DAZ *fragments (6.7 kb and 4.7 kb) with varying intensity compared to the *DAZL *fragment (Figure 1D). Quantification of the signals, both by their density and by their radioactivity, suggested that the orangutans contained various copies of *DAZ*, ranging from approximately 3 to 10 (Figure 1D, 1E; Additional file 1).

Results of our initial DAZ dosage blots using a human *DAZ/DAZL *3'UTR probe suggested the presence of only one *DAZ *gene in rhesus macaque (*Macaca mulatta*, Mmu), an Old World monkey (data not shown). This was further confirmed by careful Southern quantification using a mixture of the 3' UTRs of Mmu *DAZ *and *DAZL *as the probe and dosage standards containing known molar ratios of the 3'UTRs of Mmu *DAZ *and *DAZL*. As shown in Figure 1F, the DAZ/DAZL signal ratios of all ten rhesus monkey samples are consistent with the presence of only one *DAZ *gene.

### Conservation of the AZFc amplicons

The human *AZFc *region contains several amplicons, represented by different colors (Figure 2A, [10]). Three amplicons have been previously reported to share autosomal homology, i.e., the red-amplicon with chromosome 3, the gray-amplicon with chromosome 1, and a portion of the yellow-amplicon with chromosome 15 (Figure 2A, Table 1). We blasted the human genome with the amplicon sequences and identified additional regions with autosomal homology. A region at the proximal end of the yellow-amplicon shows 96% similarity with about 100 kb of chromosome 1 and shorter segments of several other autosomes. The green-amplicon also shares discontinuous homology with chromosomes 8 and 7. In contrast, the light-blue- and the blue-amplicons, as well as the distal portion of the yellow-amplicon lack significant homology with any autosomes or the X chromosome. To study the evolution history of *AZFc *amplicons, we identified from the various amplicons PCR fragments that detected distinguished bands on Southern blots, and hybridized them with different primate DNAs under conditions that would detect homology as low as 80% (Figure 2). The primates, in addition to the great apes studied above, included two Old World monkeys – rhesus macaque and pig-tail macaque (*Macaca nemestrina*, Mne); three New World monkeys – black-handed spider monkey (*Ateles geoffroyi*, Age), white-fronted marmoset (*Callithrix geoffroyi*, Cge), and white-faced capuchin (*Cebus capucinus*, Cca); and two prosimians – galago (with unknown species) and lemur (*Lemur catta*, Lca). Detection of male-specific fragments on Southern blots indicated the presence of homologous sequences on the Y chromosomes, and the possibility of restriction fragment length polymorphism was ruled out by using two restriction enzymes. Our results showed that blue-amplicon homologous sequences were present on the Y chromosomes of all primates except prosimians and one of the New World monkeys (Figure 2B). Sequences highly homologous to the green- and the red-amplicons were found on the Y chromosomes of all great apes and Old World monkeys, but not New World monkeys or prosimians (Figure 2C and 2D). Chromosome 15 homologous sequences on the yellow-amplicon were detected on the Y chromosomes of bonobo and chimpanzee, but not those of gorilla, orangutan, monkeys, or lower primates (Figure 2F). And finally, chromosome 1 homologous sequences in both the yellow- and the gray-amplicons were found only on the human Y chromosome (Figure 2E and 2G). Blasting the chimp genome confirmed that the chimp Y chromosome lacks these chromosome 1 homologous sequences.

**Table 1:** Evolutionary Conservation of the *AZFc *amplicons on the primate Y chromosomes

	Blue	Green	Red	Yellow		Gray
Humans						
Length	229 kb	315 kb	140 kb	678 kb		115 kb
Autosome with Homology^a^	-	8 (60 kb, 80%) 7 (21 kb, 80%)	3 (120 kb, 85%)	15 (112 kb, 95%)	1^b ^(104 kb, 96%)	1 (92 kb, 96%)
Great Apes						
*Bonobo*	+	+	+	+	-	-
*Chimpanzee*	+	+	+	+	-	-
*Gorilla*	+	+	+	-	-	-
*Orangutan*	+	+	+	-	-	-
Old World Monkeys						
*Rhesus Macaque*	+	+	+	-	-	-
*Pig-tail Macaque*	?	+	+	-	-	-
New World Monkeys						
*Spider Monkey*	+	-	-	-	-	-
*Marmoset*	-	-	-	-	-	-
*Capuchin*	+	-	-	-	-	-
Prosimians						
*Galago*	-	-	-	-	-	-
*Lemur*	-	-	-	-	-	-

a. The locations of the autosomal homologous regions within the amplicons are indicated in Figure 2A. The length and degree of homology are listed inside the parenthesis.

b. Several other autosomes also contain shorter segments of 96% homology with the same region.

## Discussion and Conclusion

Here we report our investigation on the evolution of the *AZFc *region of the Y chromosome by determining the structure and copy number of the *DAZ *gene and the conservation of *AZFc *ampliconic sequences on primate Y chromosomes. So far the genomes of three primates, i.e., human, chimpanzee, and rhesus macaque, have been sequenced [18-20], yet a completely assembled sequence of the Y chromosome is only available for the human species [10,21]. The recently published sequences of the chimpanzee Y chromosome have left out the *AZFc *equivalent amplicon-rich region [22,23], and the draft of the rhesus macaque genome came from a female [20]. Nonetheless, the NCBI database contains the sequences of several chimpanzee BAC clones that show two *DAZ *genes in the same head-to-head arrangement as their human orthologues, and our Southern results are consistent with the structural arrangements of the *DAZ *genes in these clones that encode one, two or three RRMs and various DAZ repeats.

We found significant variation in the copy number and structure organization of the *DAZ *genes in the great apes. Chimpanzees and orangutans show differences in the *DAZ *gene copy number as well as the RRM and DAZ repeat regions. In contrast, all bonobos and gorillas we studied have only 2 *DAZ *genes with polymorphism limited to the DAZ repeat region. With the limited number of animals we studied, we cannot conclude that all bonobos and gorillas have only 2 *DAZ *genes because our sampling could be biased. Studies in humans have shown that while the majority of men worldwide possess 4 *DAZ *genes, most individuals in the Y chromosome haplogroup-N as well as some in other Y-haplogroups have only two *DAZ *genes [11-14,24,25]. Previously fluorescence *in situ *hybridization (FISH) analyses of the *DAZ *genes in great apes detected one *DAZ *signal on the bonobo, chimpanzee, and gorilla Y chromosomes but two stronger *DAZ *signals on the orangutan Y chromosome [26]. Our Southern results showed the presence of more *DAZ *genes in orangutans (6 or more genes) than the other great apes (2 or 4 genes), consistent with the FISH results. The one orangutan with only 3 *DAZ *genes likely represents a rare case of partial deletion. Our finding of only one *DAZ *gene in rhesus monkey is unexpected since previous PCR amplification of exons 2–6 of rhesus macaque *DAZ *produced two sequences with Alu elements inserted at different locations [27]. Although full-length rhesus macaque *DAZ *cDNA has not yet been isolated, a *DAZ *cDNA clone of the cynomolgus monkey, another Old World monkey, contained two RRM coding regions [7]. Thus it is likely that the two previously reported exons 2–6 PCR products of rhesus macaque came from the same *DAZ *gene.

**Figure 3:**
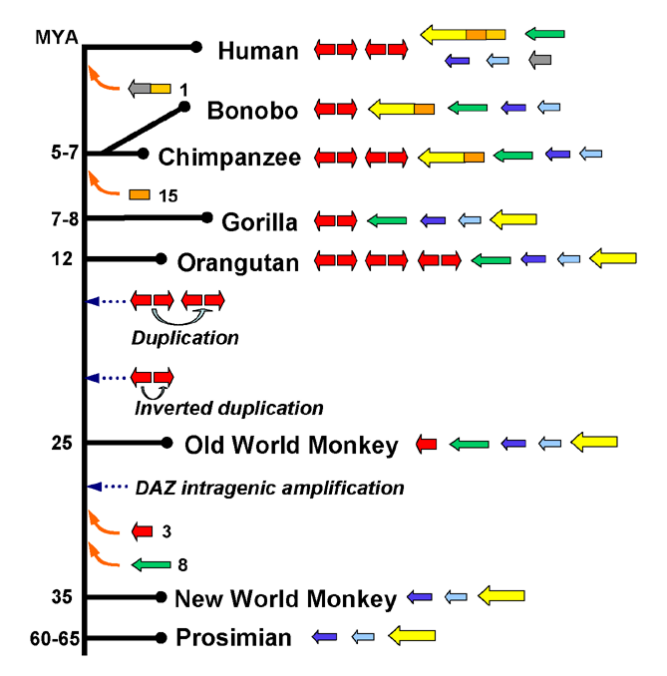
**Model of the evolution of the *AZFc *amplicons on the primate Y chromosomes**. The blue, the light-blue, and the distal portion of the yellow amplicons are the oldest amplicons and are present on the Y chromosomes of all primates. Duplication and transposition of autosomal sequences, depicted as arrows or blocks with the origin of the autosome indicated at right, to the human Y chromosome occurred in three waves as indicated with upward arrows. Today's yellow-amplicon in fact consists of three sections, represented in different colors, that arrived on the Y chromosome at different times. Additional amplifications of the red-amplicon occurred in the great ape lineage soon after the separation of the Old World monkey lineage. Subsequent duplications/deletions (not shown) in the various lineages generate a copy-number spectrum of red amplicon found in today's humans and great apes. See the text for details. The copy numbers of the gray-, green-, yellow- and blue-amplicons in the various primates have not been determined and only one each is shown. MYA: millions of years ago.

The results of our study allow us to add a few details to the existing model of the evolution of the *AZFc *region [10,21]. The lack of significant homology with any autosomes suggests that the light-blue- and the blue-amplicons as well as the distal portion of the yellow-amplicon are remnants of the ancient Y chromosome that has evolved from the same autosomal pair as the X chromosome. These amplicons thus represent the oldest members of the *AZFc *amplicons. Previous studies on the *RBMY *gene within the light-blue amplicon showed that it is conserved on the Y chromosomes of mammals and that it maintains limited homology (~75%) with the *RBMX *gene on the X chromosome [28-30]. Our detection of blue-amplicon homologous sequences on the Y chromosomes, but not autosomes or X chromosomes, of all primates higher than prosimians support the notion that the blue-amplicon has the same evolutionary origin as the light-blue amplicon [10,31]. Due to our failure of identifying PCR fragments that gave distinct bands on Southern blots, we were unable to test the origin of the distal portion of the yellow-amplicon. The remaining amplicons that maintain autosomal homology appear to have arrived on the Y chromosome in three waves (Figure 3). The first wave took place after the branching of the New World monkey lineage about 35 millions of years ago (MYA) [32] when the ancestral green- and red-amplicon sequences on separate autosomes duplicated and transposed to the Y chromosome. The sequence similarities between today's amplicons and autosomes suggest that the transposition of the green-amplicon took place much earlier than that of the red-amplicon. The second wave of duplication and transposition occurred after the branching of the gorilla lineage at 7–8 MYA and involved the chromosome 15 homologous sequence in the yellow-amplicon. And the last wave occurred after the split of the chimpanzee/bonobo and the human lineages at 5–7 MYA and involved the chromosome 1 homologous sequences in the yellow- and the gray-amplicons. It is likely that the two chromosome 1 homologous sequences were duplicated and transposed to the Y chromosome at the same time in one piece, and were separated during subsequent rearrangements into two parts that are now in different amplicons. The timings of the sequential duplication-transposition events correlate well with the sequence similarities shared between the amplicons and the autosomes in today's human genome. The red- and the green-amplicons, the first two to transpose to the Y chromosome, diverge more in sequence from the autosomes than the yellow- and the gray-amplicons which transposed more recently. It was previously suggested that the transposition of the chromosome 1 homologous sequences took place before the split of the chimpanzee and the human lineages [21]. However, our data indicate that chromosome 1 homologous sequences arrived on the Y chromosome after the two lineages separated.

The transposed autosomal sequences experienced additional changes after they arrived on the Y chromosome. As previously proposed, the ancestral *DAZL *gene in the red-amplicon underwent intragenic amplification and pruning, giving rise to the *DAZ *gene that encodes multiple copies of RRM and DAZ repeats [7,33]. Because rhesus macaque has only one *DAZ *gene, and thus one red-amplicon, we propose that at some point after the separation of the Old World monkey and the great ape lineages, the red-amplicon on the Y chromosome of an ancestor of great apes amplified once by inverted duplication to form the head-to-head arrangement observed in today's human and chimpanzee Y chromosomes [33]. Additional duplication of the head-to-head red-amplicon pair occurred sometime afterward to yield the four or more *DAZ *genes found in orangutans, chimps and humans. But when did the second duplication occur? It could have occurred independently in the orangutan, the chimpanzee, and the human lineages, but not in the gorilla and the bonobo lineages. Alternatively, it could have occurred once in an ancestor of great apes, and subsequent deletions that took place early in the bonobo and the gorilla lineages resulted in most individuals in the two species having only two *DAZ *genes. We consider the first model less likely since it requires a rare duplication event, which is not known to be promoted by long repeats, to occur three times in different lineages. On the other hand, the second model requires only one duplication event. Once there are two red-amplicon pairs on the Y chromosome, non-allelic homologous recombination between the red-amplicons on sister chromatids would result in deletion and duplication, generating *DAZ *copy number polymorphism observed in these animals [16]. Other amplicons also underwent additional changes, such as amplification, rearrangement, and acquisition of autosomal genes through retroposition [34]. The timing and the nature of these events remain to be determined.

## Methods

### Primate samples

Lymphoblastoid or fibroblast cell lines were established and blood samples were collected from great apes, monkeys and lower primates living in captivity in several zoos in Germany (Additional file 1). Peripheral blood samples were collected from several primates in the Taipei Zoo during physical examinations with the approval of the local animal committee. DNA samples of additional primates from the Los Angeles Zoo were described previously [15].

### Southern blot analysis

Genomic DNA was isolated from human and primate peripheral blood samples or cell lines using the traditional phenol extraction method. Southern blotting was carried out according to our published protocol [16]. Genomic DNA samples were digested with a restriction enzyme, separated on 0.6% agarose gels, and blotted onto nylon membrane by capillary transfer. Probe A and probe B for RRM blots and DAZ repeat blots, respectively, were PCR amplified from male chimpanzee genomic DNA [16], and the probe for DAZ dosage blots was of human origin [17]. Additional probes were PCR amplified from the various *AZFc *amplicons using a man's DNA as the template. The primer sequences (from 5' to 3') and sizes of the PCR products are: red-amplicon: F-tacatacccctcctggctg, R-ctgcacatggctcctaatc, 1.53 kb; yellow-amplicon-1: F-tactgtgattactaaactcagaag, R-ctgttgcacatttatgtacccg, 0.70 kb; yellow-amplicon-15: F-ccagttatatccccttccagc, R-gaatcttaggaagcagtctgg, 0.88 kb; gray-amplicon: F-ttgtcaaaacttgaactcacag, R-tagcagtgatattgctgatgg, 0.94 bp; green-amplicon: F-cagagaggaaagttatatcacc, R-aatcgtgagtctcgtttggac, 0.4 kb; and blue-amplicon: F-agctggaattccaacagcg, R-gacaagttgaaaccgctgg, 0.63 kb. Hybridization was carried out as previously described except that the temperature for both hybridization and washing was lowered to 60°C when samples from the monkeys and lower primates were included on the blots. After hybridization and washing, the signals of the DNA dosage blots were detected on a Typhoon 9410 variable mode imager (Amersham, Piscataway, NJ, USA) and quantified using the line analysis and graphic display programs. For the orangutan DAZ dosage blots, the hybridization bands were subsequently excised from the membranes and radioactivity counted to provide independent measurements of the signals.

### Southern blot determination of the rhesus monkey DAZ gene copy number

A 1.4 kb segment of *DAZ *3' UTR as well as the corresponding region in *DAZL *were PCR amplified from rhesus monkey genomic DNA using primers F-catgggaagttgctgcttttg and R-gttttagggatgaagccactg, cloned in the vector pCRII-TOPO (Invitrogen, Carlsbad, CA, USA) and sequenced. The 5.5 kb *DAZ *clone was linearized by digesting with BamHI, whereas the *DAZL *clone was digested with BamHI+NcoI to produce a smaller 3 kb fragment containing the gene sequence. The fragments were gel-purified and quantified. In the Southern blot analysis, the *DAZ *copy number standards contained 5 μg of BamHI digested *E. coli *DNA, 5 pg of the 3 kb *DAZL *fragment, and 4.6, 9.2, 18.3 or 27.5 pg of the 5.5 kb *DAZ *fragment, whereas each of the remaining lanes contained 5 μg of NsiI digested rhesus monkey genomic DNA. The hybridization probe contained equal moles of the 1.4 kb PCR fragments from *DAZ *and *DAZL *3'UTRs. After hybridization, the signals were quantified as described above.

## List of abbreviations

AZFc: Azoospermia factor c; DAZ: Deleted in azoospermia; DAZL: DAZ-like; RRM: RNA recognition motif; 3'UTR: 3' untranslated region; MYA: millions of years ago.

## Authors' contributions

YHY and YWL carried out the molecular analyses, JFY and WS collected the primate samples, and PHY conceived of the study and drafted the manuscript. All authors read and approved the final manuscript.

## Supplementary Material

Additional file 1 Characterization of the *DAZ *genes in great apes. The table lists the sources of the great apes and the results of the RRM and DAZ dosage blots. Click here for file
