# Supplementary material for: Evolution of the DAZ gene and the AZFc region on primate Y chromosomes
Source: BMC Evol Biol. 2008 Mar 26;8:96. doi: 10.1186/1471-2148-8-96 (PMC2322974; doi:10.1186/1471-2148-8-96)
Supplement: Additional file 1 — Characterization of the DAZ genes in great apes. The table lists the sources of the great apes and the results of the RRM and DAZ dosage blots. [file 1471-2148-8-96-S1.doc]

Supplementary Table S1. Characterization of the *DAZ* genes in great apes

| Animal Code | Sex | Source | Fragments on RRM blot (kb) | *DAZ*  copy number |
| --- | --- | --- | --- | --- |
| **Bonobo (*Pan paniscus*, Ppa)** | | | | |
| PC-F | F | Los Angeles |  |  |
| L1383 | F | Wilhelma |  |  |
| L1764 | F | San Diego |  |  |
| PC-M | M | Los Angeles | 20, 9 | 2 |
| L1509 | M | Leipzig | 20, 9 | 2 |
| L1382 | M | Wild-born | 20, 9 | 2 |
| L2466 | M | Frankfurt | 20, 9 | 2 |
| L2721 | M | Wilhelma | 20, 9 | 2 |
| 70 | M | Leipzig | 20, 9 | 2 |
| 296 | M | Wilhelma | 20, 9 | 2 |
| 481 | M | Frankfurt | 20, 9 | 2 |
| 553 | M | Antwerpen | 20, 9 | 2 |
| 561 | M | Wild-born | 20, 9 | 2 |
|  | | | | |
| **Chimpanzee (*Pan troglodytes*, Ptr)** | | | | |
| PF20 | F | Los Angeles |  |  |
| L2275 | M | Wilhelma | 31, 20 | 2 |
| L2369 | M | Wilhelma | 20 | 2 |
| 39 | M | TNO, Netherland | 31, 20, 9 | 4 |
| 594 | M | Wild-born | 20 | 2 |
| 776 | M | Wild-born | 31 | 2 |
| 794 | M | Schwabenpark | 20 | 2 |
| TZC1 | M | Taipei | 31, 20, 9 | 4 |
| TZC2 | M | Taipei | 31, 9 | 4 |
|  | | | | |
| **Gorilla (*Gorilla gorilla*, Ggo)** | | | | |
| F1728, 355 | F | Wilhelma |  |  |
| 621 | M | Wilhelma | 31, 20 | 2 |
| 673 | M | Wild-born | 31, 20 | 2 |
| 737 | M | Wilhelma | 31, 20 | 2 |
| 797 | M | Wild-born | 31, 20 | 2 |
| LA23 | M | Los Angeles | 31, 20 | 2 |
|  | | | | |
| **Orangutan (*Pongo pygmaeus*, Ppy)** B: Borneo, S: Sumatra | | | | |
| L2340, L2474 | F | Wilhelma |  |  |
| L1833 | M | Hamburg | 9 | ~6 |
| L2602 | M | Duisburg | 9 | ~6 |
| 405 (S) | M | Wild-born | Not done | ~8 |
| 407 (S) | M | Wild-born | Not done | ~6 |
| 466 (S) | M | Wilhelma | Not done | ~6 |
| 646 (S) | M | Berlin | 9 | ~8 |
| 786 (B) | M | Paris | 9 | ~6 |
| 823 (B) | M | Krefeld, Moskau | 9 | 3 |
| 827 (S) | M | Wilhelma | Not done | ~8 |
| 834 (S) | M | Duisburg | Not done | ~8 |
| 866 (B) | M | Duisburg | 9 | ~6 |
| 947 (S) | M | Studen-Zoo, CH | Not done | ~8 |
| 998 (S) | M | Munich, Hellabrunn | 9 | ~6 |
| 1005 (B) | M | Studen-Zoo, CH | Not done | ~10 |
| TZO1 | M | Taipei | 9 | ~6 |
| TZO2 | M | Taipei | 9 | ~6 |
